# Glutathione Redox System in ****β****-Thalassemia/Hb E Patients

**DOI:** 10.1155/2013/543973

**Published:** 2013-10-07

**Authors:** Ruchaneekorn W. Kalpravidh, Thongchai Tangjaidee, Suneerat Hatairaktham, Ratiya Charoensakdi, Narumol Panichkul, Noppadol Siritanaratkul, Suthat Fucharoen

**Affiliations:** ^1^Department of Biochemistry, Faculty of Medicine Siriraj Hospital, Mahidol University, Bangkok 10700, Thailand; ^2^Department of Medicine, Faculty of Medicine Siriraj Hospital, Mahidol University, Bangkok 10700, Thailand; ^3^Thalassemia Research Center, Institute of Molecular Biosciences, Mahidol University, Nakhon Pathom 73170, Thailand

## Abstract

**β**-thalassemia/Hb E is known to cause oxidative stress induced by iron overload. The glutathione system is the major endogenous antioxidant that protects animal cells from oxidative damage. This study aimed to determine the effect of disease state and splenectomy on redox status expressed by whole blood glutathione (GSH)/glutathione disulfide (GSSG) and also to evaluate glutathione-related responses to oxidation in **β**-thalassemia/Hb E patients. Twenty-seven normal subjects and 25 **β**-thalassemia/Hb E patients were recruited and blood was collected. The GSH/GSSG ratio, activities of glutathione-related enzymes, hematological parameters, and serum ferritin levels were determined in individuals. Patients had high iron-induced oxidative stress, shown as significantly increased serum ferritin, a decreased GSH/GSSG ratio, and increased activities of glutathione-related enzymes. Splenectomy increased serum ferritin levels and decreased GSH levels concomitant with unchanged glutathione-related enzyme activities. The redox ratio had a positive correlation with hemoglobin levels and negative correlation with levels of serum ferritin. The glutathione system may be the body's first-line defense used against oxidative stress and to maintain redox homeostasis in thalassemic patients based on the significant correlations between the GSH/GSSH ratio and degree of anemia or body iron stores.

## 1. Introduction

*β*-thalassemia/Hb E is the most prevalent form of *β*-thalassemia and hemoglobinopathy in Southeast Asia, consisting of *β*-thalassemia and Hb E (substitution of glutamic acid by lysine at position 26 of the *β*-globin chain) alleles on chromosome 11. These quantitative and qualitative defects of *β*-globin synthesis result in an excess of unmatched *α*-globins, which release free iron, nonheme iron, or hemichrome [1]. These iron species catalyze Fenton reaction leading to overproduction of reactive oxygen species (ROS) in erythrocytes especially hydroxyl radicals and superoxide anions, as has been reported in various studies [2–4]. To protect or at least minimize the damage to cells from radical-induced oxidative damage, the body has defense systems consisting of enzymatic and nonenzymatic antioxidants either obtained from the diet or from self-synthesis.

Reduced glutathione (*γ*-L-glutamyl-L-cysteinylglycine, GSH) is the most abundant thiol-containing peptide existing in most cell types, especially liver, spleen, kidneys, erythrocytes, and lens of the eye. It is the key molecule for maintenance of cellular redox because of its strong electron-donation potential via the sulfhydryl groups of cysteine residues (*E*_0_′ = −0.33 V) and its high concentration in cells [5]. Under oxidative stress, GSH donates reducing equivalents to free-radical scavenging enzymes, including glutathione peroxidase (GPx) and glutathione-S-transferase (GST), and converts to its oxidized form (GSSG). This GSSG can be reconverted to GSH by a reaction catalyzed by glutathione reductase (GR). Therefore, a lower ratio of reduced to oxidized glutathione (GSH/GSSG) may indicate higher oxidative stress in cells. To date, the ratio has been widely used as an indicator of cellular redox status in various diseases with enhanced oxidative stress [6–8]. In this study, blood profiles of total, reduced, and oxidized glutathione were analyzed using the HPLC method with fluorometric detection. The GSH/GSSG ratios were then calculated concomitant with measuring the activities of glutathione-related enzymes (GPx, GST, and GR) and serum ferritin levels in nonsplenectomized and splenectomized *β*-thalassemia/Hb E patients compared to normal subjects. 

## 2. Materials and Methods

### 2.1. Subjects and Blood Sampling

Twenty-five patients with heterozygous *β* ^0^-thalassemia and Hb E hemoglobin typing (*β* ^0^ *β* ^E^) were recruited from the Division of Hematology, Department of Medicine, Faculty of Medicine Siriraj Hospital, Mahidol University, Thailand. Inclusion criteria for all patients were age between 18 and 50 years, hemoglobin concentration 60–90 g/L, no blood transfusion, no drugs administered for at least 3 months before blood sampling, aspartate aminotransferase (AST) or alanine aminotransferase (ALT) ≤ 3 times the upper limit of normal range, and creatinine clearance ≥ 10 mL/min. Twenty-seven normal subjects age- and sex-matched with patients were recruited as the control group. The healthy volunteers had normal hemoglobin typing (*A*_2_ *A*) and normal values of hematological parameters. All subjects were nonsmoking, did not drink alcohol, and no drugs were administered within 1 month prior to the study. They signed informed consents and none of the females were in pregnancy or lactation. The protocol was approved by the Siriraj Institutional Review Board, Mahidol University, Thailand. 

### 2.2. Blood Collection

Eight milliliters of fasting blood were individually withdrawn and collected in an EDTA tube (5 mL) and a plain tube (3 mL). Two milliliters of EDTA blood were measured for hematological parameters using the Sysmex NE-1500 hematology analyzer (TOA Medical Electronics Co., Ltd., Kobe, Japan) and for glutathione analysis by HPLC with fluorescence detector. The remainder (3 mL) was centrifuged at 800 ×g for 10 minutes at 4°C to remove the plasma and buffy coat. The red cells were washed a further 3 times with cold phosphate buffered saline (pH 7.4) and adjusted to a 50% red cell suspension for assays of enzymatic activities. Serum from the clotted blood (3 mL) was prepared by centrifugation at 800 ×g for 10 minutes at 4°C and analyzed for serum ferritin using an automated analyzer Integra 700 (Roche Diagnostics, Basel, Switzerland).

### 2.3. Measurement of Total, Reduced, and Oxidized Glutathione in Blood

Various forms of blood glutathione in normal subjects and *β*-thalassemia/Hb E patients were analyzed using the HPLC method with fluorometric detection according to the method of Pastore et al. [9]. This technique is based on derivatization of thiol groups with bromobimane to yield highly fluorescent thioethers that can be detected in a fluorescence detector during elution.

#### 2.3.1. Sample Preparation

Briefly, each fresh blood sample was transferred into 2 Eppendorf tubes; one to measure total glutathione (GSH + GSSG) and another is just its oxidized form (GSSG). Then, 10 mM phosphate buffer (pH 7.2) and 1 M N-ethylmaleimide (NEM) were immediately mixed into the GSH + GSSG and GSSG tubes, respectively. After hemolysis with cold water, 12% sulfosalicylic acid was added to precipitate proteins and then the tubes were centrifuged at 10,000 ×g for 5 minutes to obtain the supernatant.

#### 2.3.2. Glutathione Derivatization

 Freshly prepared 50 *μ*M cysteamine was used as internal standard. The mixture containing internal standard and supernatant (1 : 1) was added with a prederivertized solution consisting of 2 mM EDTA-dithiothreitol (DTT), 4 M NaBH_4_, 1-octanol, and 1.8 M HCl to maintain thiol groups in a reduced state and protect against foaming. After incubating for 3 minutes, 1.5 M *N-*ethylmorpholine buffer (pH 8.0) and 25 mM bromobimane were added and incubated for additional 3 minutes. Acetic acid was finally added to terminate the reaction.

#### 2.3.3. Chromatography

The HPLC system used in this study was an Alliance HPLC 2695 Separation Module (Waters Corporation, MA, USA) and the detector was a Waters 2475 Multi Wavelength Fluorescence Detector (Waters Corporation, MA, USA). A Hypersil-ODS analytical column (150 × 4.6 mm, 3 *μ*M particle size with a Hypersil-BDS C18 guard column; Thermo Electron Corporation, OH, USA) was equilibrated with 30 mM ammonium nitrate and 40 mM ammonium formate buffer (pH 3.6, buffer A) for 10 minutes. The separation of thiols was performed at a flow rate of 1.5 mL/minute with a 6-minute gradient of acetonitrile (buffer B), starting with 0–30% buffer B for initial 4 minutes, then 30–100% buffer B for 1 minute, and ending with 100% buffer B for 1 minute. The fluorescence detector was set at 390 nm excitation and 478 nm emission wavelengths. A 20 *μ*L derivatized sample was injected into the chromatographic system. In this system, the glutathione standard and 50 *μ*M cysteamine were eluted at 3.75 and 4.18 minutes. Glutathione and the internal standard eluted at the same retention times in whole blood from all normal subjects and *β*-thalassemia/Hb E patients.

#### 2.3.4. Calculation of Glutathione Levels

 The data were analyzed with Empower chromatography data software (Waters Corporation, MA, USA). The linearity of the calibration curve was assessed in the range of 0–100 *μ*M glutathione and the limit of detection was about 50 nM. Total glutathione (GSH + GSSG) and oxidized glutathione (GSSG) levels were obtained from samples with and without NEM added, respectively. The GSH level was calculated by subtraction of the GSSG level from the total glutathione.

### 2.4. Measurement of Glutathione S-Transferase Activity

Assay of total glutathione S-transferase (GST) was based on the GST-catalyzed conjugation of 1-chloro-2,4-dinitrobenzene (CDNB) with GSH [10]. The reaction generates a dinitrophenyl thioether that can be detected by a spectrophotometer at 340 nm; increases in absorption are proportional to the GST activity in the sample. The GST activity was reported in international units per milligram of hemoglobin (U/mg Hb) with an extinction coefficient of 0.0096 *μ*M^−1^ cm^−1^ at 340 nm.

### 2.5. Measurement of Glutathione Reductase Activity

Glutathione reductase (GR) catalyzes the NADPH-dependent reduction of GSSG to GSH. Assay of GR activity was based on the method of Andersen et al. [11], in which all reagents except GR were provided in excess. Therefore, the decreased value of *A*_340_ associated with NADPH oxidation was directly proportional to the GR activity in the sample. The GR activity was reported in international units per milligram of hemoglobin (U/mg Hb) with an extinction coefficient for NADPH of 0.00622 *μ*M^−1^ cm^−1^ at 340 nm.

### 2.6. Measurement of Glutathione Peroxidase Activity

Glutathione peroxidase (GPx) catalyzes the reduction of hydroperoxide using GSH as a reducing equivalent, and glutathione in oxidized form (GSSG) can be recycled to the reduced form (GSH) catalyzed by GR coupled with NADPH oxidation. The activity of GPx in red cells was determined by the method of Beutler [12]. Similar to the GR assay, all reagents except GPx were provided in excess and *t*-butyl hydroperoxide was used as the substrate. Thus, the decreased value of *A*_340_ caused by NADPH oxidation was proportional to the GPx activity in the sample. The GPx activity was reported in international units per gram of hemoglobin (U/g Hb) with an extinction coefficient for NADPH of 0.00622 *μ*M^−1^ cm^−1^.

### 2.7. Statistical Analysis

The data were analyzed using the two-tailed nonparametric Mann-Whitney test to determine the differences between groups. Spearman's correlation coefficient was used to analyze correlations between two variables. *P* values less than 0.05 were considered statistically significant.

## 3. Results

The hematological parameters of 27 normal subjects (13 males and 14 females) and 25 *β*-thalassemia/Hb E patients (12 males and 13 females) are summarized in Table 1. All hematological parameters in patients were significantly different from normal subjects (*P* < 0.001, except *P* = 0.015 for WBC count and 0.035 for platelet count). Among the patients, 8 splenectomized (SP) patients (5 males and 3 females) had significantly higher WBC counts, MCVs, platelet counts, and reticulocyte percentages than observed in 17 nonsplenectomized (NS) patients (7 males and 10 females) (*P* < 0.001). The MCHC levels in SP patients were significantly lower than in the NS group (*P* < 0.001), whereas other red cell parameters including hemoglobin concentration and red cell count were not statistically different between the two groups.

Whole blood collected from all subjects was used to analyze total glutathione, GSH, GSSG, and GSH/GSSG ratio; meanwhile, glutathione-related enzymes (GST, GR, GPx) in red blood cells and ferritin in serum were also analyzed (Table 2). Their significantly higher level of serum ferritin (3255 ± 482 pmol/L, *P* < 0.001) suggests iron loading in the patients. Concentrations of total glutathione were significantly lower in patient whole blood (607.1 ± 39.3 *μ*mol/L) in comparison with normal subjects (896.0 ± 46.2 *μ*mol/L, *P* < 0.001). Decreased levels of GSH were found in patients (285.2 ± 26.3 *μ*mol/L) compared to normal subjects (804.4 ± 42.0 *μ*mol/L, *P* < 0.001) accompanied by higher GSSG levels in patients (321.9 ± 18.6 *μ*mol/L) than in the normal group (91.6 ± 6.4 *μ*mol/L, *P* < 0.001). Therefore, the GSH/GSSG ratio in *β*-thalassemia/Hb E patients (0.9 ± 0.1) was significantly lower than in normal controls (9.5 ± 0.6, *P* < 0.001). Patients showed significantly higher activities of GST, GR, and GPx compared to normal subjects (*P* < 0.001).

**Figure 1:**
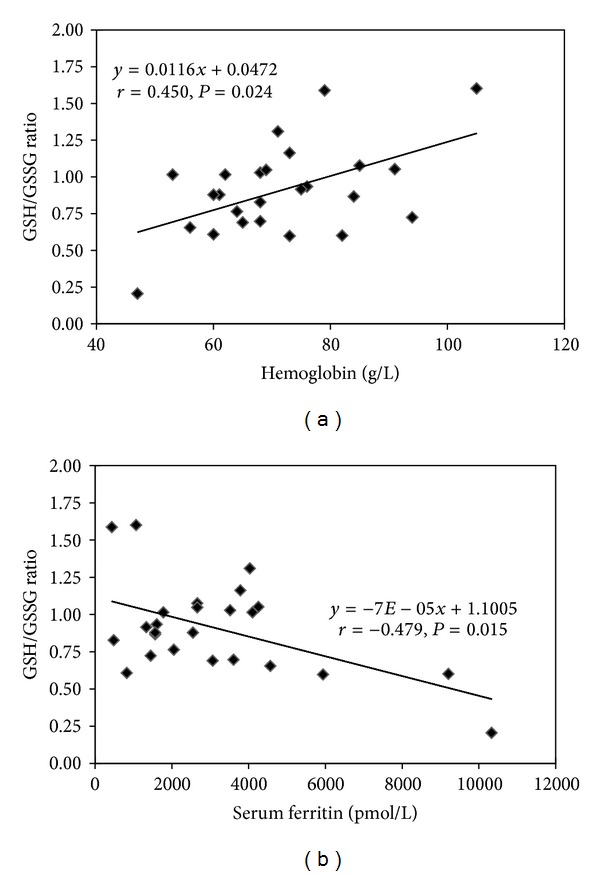
Linear regression analysis in *β*-thalassemia/Hb E patients. (a) Correlation between hemoglobin level and GSH/GSSG ratio. (b) Correlation between serum ferritin level and GSH/GSSG ratio.

Levels of serum ferritin were significant higher in SP patients (5156 ± 1112 pmol/L) in comparison with NS patients (2361 ± 319 pmol/L, *P* = 0.004). Splenectomized individuals had significantly decreased levels of total glutathione and GSH as compared to the NS group (*P* = 0.033 and 0.020, resp.). Although no statistical difference was found in GSSG levels, the GSH/GSSG ratio in SP (0.7 ± 0.1) was significantly lower than in NS (1.0 ± 0.1, *P* = 0.024). Moreover, the activities of GST, GR, and GPx in RBC did not show significant differences between the two groups. Correlation analyses revealed a significantly positive correlation between GSH/GSSG ratio and hemoglobin concentration (*r* = 0.450, *P* = 0.024) and a negative correlation between GSH/GSSG ratio and serum ferritin (*r* = −0.479, *P* = 0.015), as shown in Figure 1.

## 4. Discussion

Anemia in thalassemia is the combined result from ineffective erythropoiesis of erythroid progenitor cells and peripheral hemolysis of matured red blood cells [1]. The critical mechanism underlying the anemia is thought to be ROS overproduction catalyzed by iron-induced Fenton or Haber-Weiss reactions. Oxidant injury to membrane proteins causes cytoskeletal abnormalities including reduced spectrin/band 3 ratio, partial oxidation of band 4.1, phosphatidylserine (PS) exposure, and clustering of band 3 [13], leading to RBC deformity and complement-mediated erythrophagocytosis [14]. The hyperhemolysis state was reflected in low levels of hemoglobin at steady state and compensation for anemia was shown by an approximately 6-fold increase in the percentage of reticulocytes in *β*-thalassemia/Hb E patients. Splenectomized patients had 3-fold more platelets and 10-fold higher WBC levels than the NS group. This may be explained by virtue of the fact that the spleen is the organ removing aging or abnormal RBC, foreign invaders, and other moieties including platelets, WBCs, or even PS-exposed RBCs from the circulation. Loss of the spleen raises circulating platelets and WBC, which may contribute to a high risk of thrombosis and certain infections including meningitis, pneumonia, and sepsis [15, 16]. Although we did not find a significant difference in Hb concentration when comparing SP and NS patients, an approximately 5-fold higher reticulocyte count in the SP group was reported in this study, suggesting the role of spleen in reticulocyte pooling [17].

Thalassemic patients undergo chronic oxidative stress and prooxidant pools, evidenced by high levels of serum iron, ferritin, and nontransferrin bound iron [18–20]. Many studies have reported on increased levels of oxidative products of biomolecules including malondialdehyde (MDA), protein carbonyl, and 8-hydroxyguanine in patients with thalassemia [18, 21, 22]. Reduced glutathione is one of the most important intracellular and extracellular antioxidants, synthesized from its amino acid constituents (cysteine, glutamic acid, and lysine) by two sequential enzymes, *γ*-glutamylcysteine synthetase (*γ*-GCS) and glutathione synthetase. The rate-limiting step in GSH synthesis is the formation of *γ*-glutamylcysteine, catalyzed by *γ*-GCS [23]. Glutathione serves as an antioxidant by direct radical scavenging or participating in enzymatic reactions. Consequently, GSH is oxidized to GSSG, which can be converted back to GSH in a reaction catalyzed by NADPH-dependent GR.

The GSH/GSSG ratio is considered a sensitive index of redox status since its concentration is far higher than those of other cellular redox systems including the adenine dinucleotide phosphate system (NADPH/NADP^+^) and the thioredoxin couplet (Trx (SH)_2_/TrxSS), with intracellular and extracellular concentrations of glutathione in the range of 0.5–10 mM and 2–20 *μ*M, respectively [5]. Disturbance of GSH redox status affects various biological processes including activation of redox-sensitive transcriptional factors, redox-regulated signaling pathways, inflammation, apoptosis, and cell proliferation [23]. Decreased values of this ratio have been reported in many diseases associated with increased oxidative stress, including cancer [24–26], diabetes [27], aging [28], HIV infection [29], and cardiovascular diseases [6, 30].

In this study, whole blood from patients with *β*-thalassemia/Hb E showed a 90% reduction in the GSH/GSSG ratio compared to controls, suggesting reduced GSH availability and increased GSSG accumulation. The remarkably increased GSSG in thalassemic patients was likely due to cellular GSH overutilization, supported by 123% and 93% increases in GST and GPx activities, respectively. The GST enzyme detoxifies xenobiotics, including metabolites from oxidative reactions, by conjugating with GSH, while GPx is an antioxidant enzyme that reduces hydrogen peroxide to water using GSH as a reducing equivalent. Recycling of GSH by a 67% increase in GR activity was still unable to handle the increased GSH demand. The SP patients had significantly decreased GSH with a nonsignificant change in GSSG levels compared to the NS group. The significantly lower GSH/GSSG in SP patients revealed higher iron-induced oxidative stress, supporting previous studies where levels of transferrin saturation, plasma nontransferrin-bound iron, and serum ferritin were found to be significantly higher in SP **β**-thalassemia/Hb E patients than in the NS group [31, 32].

In thalassemia, hemoglobin concentration is related to the amount of unmatched globins [33]. A low Hb concentration implies a high level of unmatched globins, followed by high levels of iron-induced oxidative stress and activated antioxidant defense. Our results showed a positive correlation between Hb concentration and the GSH/GSSG ratio, while Hb levels did not correlate with GST, GR, and GPx activities in red cells. Thus, glutathione may be the first line of protection against oxidative stress in thalassemic erythrocytes, while the increased activities of such enzymes is induced by excess oxidants or secondary oxidative metabolites such as aldehydes and ketones [32]. Moreover, the GSH/GSSG ratio also negatively correlated with serum ferritin levels indicating the role of iron in inducing oxidative stress in thalassemic patients.

The intracellular GSH level depends on levels of GSH synthesis, efflux, utilization, and recycling. In our study, GSH utilization (GST and GPx) and recycling (GR) did not show significant differences among thalassemic patients. Therefore, the status of the redox environment in thalassemia is mainly regulated by the rate of GSH export and GSH synthesis. Oxidative stress-induced GSH efflux through membrane transporters in the families of cystic fibrosis transmembrane conductance regulator (CFTR) and multidrug resistance-associated protein (MRP) were reported in *β*-thalassemia/Hb E erythrocytes [34]. Strategies aimed at minimizing GSH efflux or maximizing GSH synthesis should be emphasized that include administration of CFTR/MRP inhibitors [34], supplementation of cysteine-donating compounds (a rate-limiting substrate) such as N-acetyl-L-cysteine (NAC) and nacystelyn (NAL), or providing GSH-esters which bypass feedback inhibition of the *de novo* GSH synthesis [23]. Dietary polyphenols, such as resveratrol, induce GSH synthesis by activating nuclear erythroid-related factor 2 (Nrf2), followed by increased expression of the rate-limiting enzyme *γ*-GCS [35]. Nutritional or medicinal knowledge of the regulation of GSH metabolism and maintenance of GSH homeostasis are important for alternative treatments to improve the health of thalassemic patients.

## Figures and Tables

**Table 1:** Hematological parameters of normal and *β*-thalassemia/Hb E subjects (mean ± SEM).

Subjects	Hematological data							
	Hb	WBC	RBC	MCV	MCH	MCHC	Platelet	Reticulocyte
	(g/L)	(×10^9^ cells/L)	(×10^12^ cells/L)	(fL)	(pg)	(g/L)	(×10^9^ cells/L)	(%)
Normal control (*n* = 27)	143.6 ± 3.0	6.2 ± 0.3	4.9 ± 0.1	88.4 ± 0.5	28.6 ± 1.0	334.7 ± 0.2	251.8 ± 8.7	1.1 ± 0.1
*β*-thalassemia/Hb E (*n* = 25)	71.6 ± 2.7	30.9 ± 10.1	3.8 ± 0.2	60.2 ± 1.7	19.0 ± 0.5	316.5 ± 0.4	359.0 ± 50.6	6.4 ± 1.6
*P* value	<0.001	0.015	<0.001	<0.001	<0.001	<0.001	0.035	<0.001

Splenectomized (*n* = 8)	70.6 ± 6.3	79.8 ± 24.4	3.5 ± 0.4	67.8 ± 2.6	20.2 ± 0.9	298.5 ± 0.5	656.6 ± 76.2	14.1 ± 3.9
Nonsplenectomized (*n* = 17)	72.0 ± 2.8	7.9 ± 0.7	3.9 ± 0.1	56.6 ± 1.4	18.4 ± 0.5	324.9 ± 3.2	214.3 ± 18.9	2.7 ± 0.3
*P* value	0.817	<0.001	0.314	<0.001	0.062	<0.001	<0.001	<0.001

Hb: hemoglobin; MCH: mean corpuscular hemoglobin; MCHC: mean corpuscular hemoglobin concentration; MCV: mean corpuscular volume; RBC: red blood cells; WBC: white blood cells.

**Table 2:** Serum ferritin, glutathione concentration, redox ratio (GSH/GSSG), and GSH-related enzyme activities (GST, GR, GPx) of normal subjects and *β*-thalassemia/Hb E patients (mean ± SEM).

Subjects	Serum	Glutathione and GSH-related enzymes							
	ferritin	Hb	Total GSH	GSH	GSSG	GSH/GSSG	GST	GR	GPx
	(pmol/L)	(g/L)	(*µ*mol/L)	(*µ*mol/L)	(*µ*mol/L)		(U/mg Hb)	(U/mg Hb)	(U/g Hb)
Normal subjects (*n* = 27)	174 ± 14	143.6 ± 3.0	896.0 ± 46.2	804.4 ± 42.0	91.6 ± 6.4	9.5 ± 0.6	5.6 ± 0.5	4.6 ± 0.2	31.5 ± 1.5
*β*-thalassemia/Hb E (*n* = 25)	3255 ± 482	71.6 ± 2.7	607.1 ± 39.3	285.2 ± 26.3	321.9 ± 18.6	0.9 ± 0.1	12.5 ± 0.8	7.7 ± 0.6	60.7 ± 2.2
*P* value	<0.001	<0.001	<0.001	<0.001	<0.001	<0.001	<0.001	<0.001	<0.001

Splenectomized (*n* = 8)	5156 ± 1112	70.6 ± 6.3	487.2 ± 54.0	198.2 ± 48.6	288.9 ± 26.3	0.7 ± 0.1	10.4 ± 1.6	8.9 ± 1.2	58.8 ± 4.6
Nonsplenectomized (*n* = 17)	2361 ± 319	72.0 ± 2.8	663.5 ± 46.8	326.1 ± 26.7	337.4 ± 23.9	1.0 ± 0.1	13.5 ± 0.9	7.1 ± 0.7	61.6 ± 2.5
*P* value	0.004	0.817	0.033	0.020	0.231	0.024	0.079	0.186	0.577

GPx: glutathione peroxidase; GR: glutathione reductase; GSH: glutathione; GSSG: glutathione disulfide; GST: glutathione-S-transferase.
